# Influence of Kazakhstan’s Shungites on the Physical–Mechanical Properties of Nitrile Butadiene Rubber Composites

**DOI:** 10.3390/polym16233370

**Published:** 2024-11-29

**Authors:** Kanat Beknazarov, Rustam Tokpayev, Abdirakym Nakyp, Yulia Karaseva, Elena Cherezova, Miroslawa El Fray, Svetoslav Volfson, Mikhail Nauryzbayev

**Affiliations:** 1 Center of Physical Chemical Methods of Research and Analysis, Faculty of Chemistry and Chemical Technology, Al-Farabi Kazakh National University, Al-Farabi Ave. 71, Almaty 050038, Kazakhstan; kanat.beknazarov@kaznu.edu.kz (K.B.); rustamtokpaev@mail.ru (R.T.); nauryzbaev@cfhma.kz (M.N.); 2 Institute of Polymers, Kazan National Research Technological University, 68 K. Marx Str., 420015 Kazan, Russia; karaseva_j@mail.ru (Y.K.); cherezova59@mail.ru (E.C.); svolfson@kstu.ru (S.V.); 3 Department of Polymer and Biomaterials Science, Faculty of Chemical Technology and Engineering, West Pomeranian University of Technology in Szczecin, Al. Piastów 45, 70-311 Szczecin, Poland; mirfray@zut.edu.pl

**Keywords:** shungite ore, flotation enrichment, shungite concentrate, carbon black, acrylonitrile butadiene rubber, vulcanization, physical–mechanical properties

## Abstract

This study presents data on the use of shungite ore (the Bakyrchik deposit, Kazakhstan) and its concentrate as fillers in elastomer composites based on nitrile butadiene rubber. In addition to carbon, these shungite materials contain oxides of Si, Fe, K, Ca, Ti, Mn, and Al. The shungite concentrate was obtained through a flotation process involving five stages. The chemical composition analysis of these natural fillers revealed that during flotation, the carbon content increased 3.5 times (from 11.0 wt% to 39.0 wt%), while the silicon oxide content decreased threefold (from 49.4 wt% to 13.6 wt%). The contents of oxides of K, Ca, Ti, Mn, and Al decreased by less than 1%, and iron oxide content increased by 40% (from 6.7 wt% to 9.4 wt%). The study explored the impact of partial or full replacement of carbon black (CB) of P 324 grade with the shungite ore (ShO) and the shungite concentrate (ShC) on the vulcanization process and the physical–mechanical properties of the rubber. It was found that replacing CB with ShO and ShC reduces Mooney viscosity ML (1 + 4) 100 °C of the rubber compounds by up to 29% compared to the standard CB-filled sample. The use of the shungite fillers also increased scorch time (t_s_) by up to 36% and cure time (t_90_) by up to 35%. The carbon content in the shungite fillers had little influence on these parameters. Furthermore, it was demonstrated that replacing 5–10 wt% of CB with ShO or ShC improves the tensile strength of the rubber. The results of the flotation enrichment process enable the assessment of how these shungite fillers affect the properties of the composites for producing rubbers with specific characteristics. It was also found that substituting CB with ShO or ShC does not significantly affect the rubber’s resistance to standard oil-based media. The findings indicate that Kazakhstan’s shungite materials can be used as fillers in rubber to partially replace CB.

## 1. Introduction

Shungite rocks can serve as a source for obtaining carbon-containing materials. Shungite is found in Russia, Ukraine, India, the Congo, the USA, Austria, and Kazakhstan [1].

Shungite rocks are a unique natural composite in composition and structure, composed of amorphous carbon and highly dispersed crystalline silicate material. Based on the composition of the mineral matrix, shungites are classified into siliceous, aluminosilicate, and carbonate varieties [2]. Shungite carbon is uniformly distributed within a silicate framework of fine-dispersed quartz crystals [3,4].

**Figure 1:**
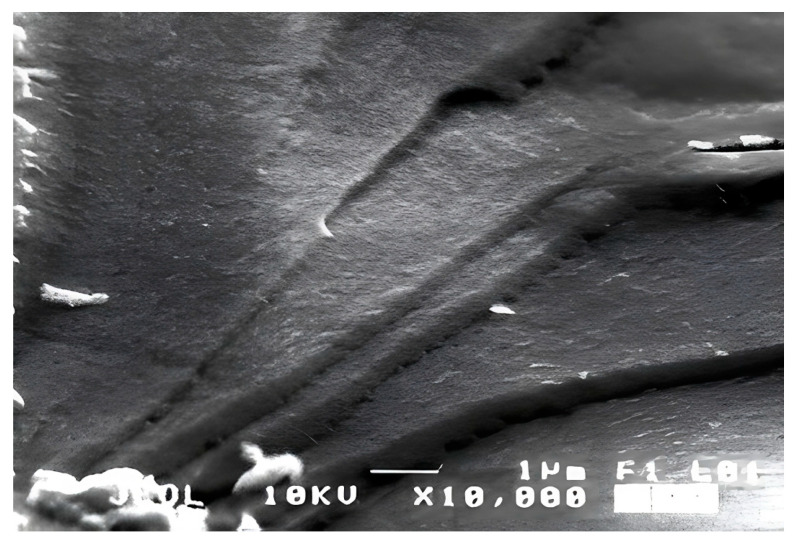
SEM images of the stepped micromorphology of fractures in high-grade anthraxolites from Kazakhstan.

**Figure 2:**
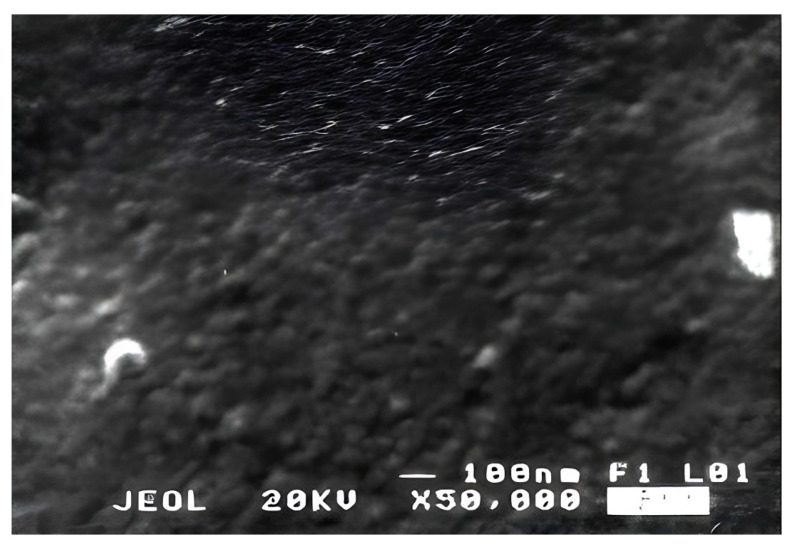
High-resolution SEM images of the globular structure of Kazakhstan’s anthraxolite.

Shungite rocks from the deposits in Eastern Kazakhstan (the Bakyrchik deposit), like those from the Zazhoginsky deposit in Karelia (Russia), are classified as anthraxolites (Figure 1 and Figure 2) [5,6].

Anthraxolites are a natural system of solid bitumens, whose chemical characteristics vary widely depending on the mineral phase and the degree of metamorphism. Anthraxolite contains up to 30 wt% of an allotropic form of metastable carbon in the pre-graphite stage of carbonification, with fullerene-like regular structures comprising up to 0.001 wt% [7]. Kazakhstan’s shungite (the Bakyrchik deposit, 19.1 wt% of carbon) exhibits a turbostratic structure, similar to carbon black (CB) crystallites but with more disordered carbon. It is characterized by an interlayer spacing of approximately 0.35 nm, akin to shungite from the Zazhoginsky deposit in Russia [8,9,10].

Shungite rocks are of interest as sorbent materials for the purification of wastewater from petroleum products and phenols. Shungite possesses a broad spectrum of bactericidal properties; it exhibits adsorptive activity toward certain bacterial cells, phages, and pathogenic saprophytes [11].

Shungite from the Bakyrchik deposit is known to be used as a sorbent for oil extraction and determination of synthetic dyes in liquids [12,13,14,15,16,17], as well as electrode materials [18] and carriers of carbon–metal catalysts [19].

Crystals of crushed, finely ground shungite exhibit pronounced bipolar properties. As a result, shungite has a high level of adhesion and the ability to mix with virtually all organic and inorganic substances. This property allows shungite to be used as a filler for polymer and other composite materials [20,21,22].

In the work [23], elastomer compositions based on acrylonitrile butadiene rubber of BNR-40 grade and polybutadiene rubber BR with the addition of shungite (carbon content 28–32 wt%) were described. The authors [24,25,26,27] found that the addition of ground shungite filler (carbon content 30–33 wt%) gives elastomer composites increased strength properties and frost resistance.

The addition of shungite fillers in the manufacture of latex gloves makes it possible to obtain products with properties characteristic of gloves manufactured by standard technology [28]. In the study [29], shungite (carbon content 20–30 wt%) with a specific surface area of 20 m^2^/g was used in rubber compounds. It has been shown that the introduction of shungite into rubber compounds reduces the rubber content in the bitumen-rubber binder system without a loss of quality [30].

A streamlined method to covalently bond hydroxylated carbon nanotubes (CNOH) within a polyphenol matrix, achieved through a direct, solvent-free process, was presented in paper [31]. This allows achieving a significant improvement in the mechanical properties of composites using low concentrations of CNOH along with topologically contrasting linkers.

In [32], the authors investigated the effects of interactions between nitrile butadiene rubber (NBR) and silica on the development of agglomerates by silica particles and of bound rubber in NBR/silica composites. The study showed that hydrogen bonding between nitrile and silanol groups suppresses the development of agglomerates by silica particles and affects the mechanical properties of NBR/silica composites by reducing agglomeration and improving strength properties.

The authors of works [33,34] used shungite to produce oil–petrol- and seawater-resistant rubbers. Studies [35,36,37,38,39] have shown that nanostructured shungite materials can increase the shielding efficiency of electromagnetic shielding composites due to their high absorption coefficient, as well as their ability to interact with the thermoplastic matrix, improving their thermophysical and electrophysical properties. The proposed composites have high chemical and thermal resistance.

Shungite rocks remain in the dumps of several deposits after the mining and enrichment of polymetallic ores. These shungite rocks are subjected to carbon enrichment and activation (thermal, mechanochemical) to increase the specific surface area and pore volume [40,41]. The concentrates can then be used as sorbents and fillers.

In particular, the concentrate of Kazakhstan’s shungite (carbon content 45 wt%), obtained through flotation enrichment for carbon, is used as a sorbent for the capture of organic compounds and CO_2_, demonstrating high speed and good selectivity [42,43].

Carbonate shungite of the ”Taurite” grade (“Koksu” mining company” LLP, Kazakhstan) was used as a filler for bitumen after mechanochemical activation [44]. A “green” method for obtaining graphene from shungite is also known [45]. The synthesis of carbon materials from wastes of various industries fits into the paradigm of promising models of “clean energy” development.

After the mining of polymetallic ores at the Bakyrchik gold-sulfide deposit, three types of shungite-bearing rocks remain in the dumps. The main type is characterized as medium-carbon shungite (5–25% grade).

This work describes the method of obtaining shungite concentrate (ShC) by flotation enrichment to increase the carbon content from shungite ore (ShO) of the Bakyrchik deposit. The purpose of this work is to study the possibility of using ShO and ShC as a filler for rubber as an alternative to carbon black.

From the literature data presented, it follows that natural amorphous-crystalline carbon-silicate composites of various deposits (shungite ores), containing a maximum of 28 wt% of carbon, are successfully used as fillers for polymer materials. At the same time, CB is partially replaced by shungite. Shungite increases the plasticity and operational properties of rubbers and improves the technological characteristics of rubber compounds.

Replacing CB with shungite in rubber compositions is economically feasible because shungite has a lower cost compared to CB, allowing for a reduction in the total cost of obtaining polymer composites. In addition, the use of shungite improves the sanitary and hygienic conditions at the factories, as shungite is less “dusty” compared to CB.

The novelty of this work is that, for the first time, the shungite ore from the Bakyrchik deposit in the Abay Region, which is related to anthraxolites, and its concentrate enriched with carbon obtained by the flotation method, were investigated as fillers replacing carbon black in elastomer composites based on nitrile butadiene rubber. In this case, it was believed that increasing the carbon content of the shungite filler would improve the physical–mechanical and operational properties of rubber.

## 2. Materials and Methods

### 2.1. Materials

Nitrile butadiene rubber of BNKS-28 AMN grade (technical specifications TU 38.30313-2006) (2nd group, Krasnoyarsk Synthetic Rubber Plant JSC, Krasnoyarsk, Russia) was used in this work. Technical sulfur (1st class, 9995 grade, SERA CJSC, Orenberg, Russia) was used as a vulcanizing agent. The vulcanization accelerator 2-mercaptobenzothiazole (MBTS) (1st grade, Volzhsky Orgsintez JSC, Volgograd, Russia) was used. The vulcanization activator zinc oxide (ZnO) (A grade, Empils-Zinc LLC, Rostov-on-Don, Russia) and stearic acid (T-32 grade, Nefis Cosmetics JSC, Kazan, Russia) were used. The fillers included CB of P 324 grade (Sterlitamak Petrochemical Plant JSC, Sterlitamak, Russia), ShO (the Bakyrchik deposit, Almaty, Kazakhstan), and ShC obtained by flotation enrichment from ShO. Carbon Tetrachloride (CCl_4_) was used as the solvent (State Standard GOST 20288-74, EKOS-1 JSC, Moscow, Russia). Kerosene TS-1 and pine oil Flotol 5219 (Clariant, Muttenz, Switzerland) were used as flotation reagents. The petroleum liquid of SZhR-1 grade was used as the standard hydrocarbon media in determining the properties of rubbers and rubber-technical products (technical specifications TU 38 10195-86, Gazpromneft-Omsk Refinery JSC, Omsk, Russia).

### 2.2. Preparation of Shungite Fillers

The preliminary preparation of shungite fillers was carried out on a grinding complex (GC) by grinding shungite ore to powder with particle sizes of 1–20 μm.

**Figure 3:**
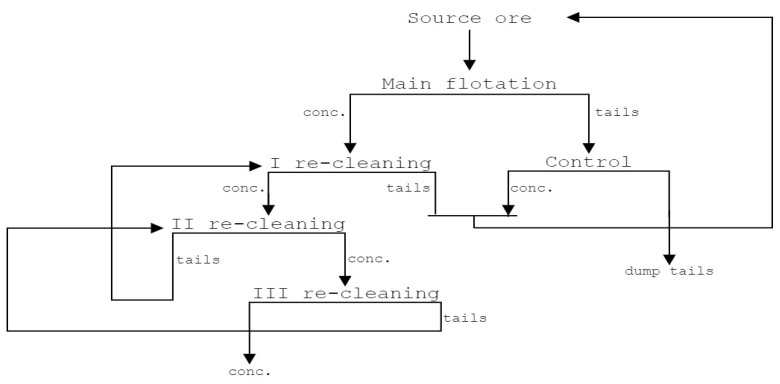
Technological scheme of flotation enrichment of the shungite ore from the Bakyrchik deposit.

Flotation enrichment of the shungite ore was carried out in a laboratory flotation unit FML-3 (NPK Mechanobr-Technika, St. Petersburg, Russia) with a chamber volume of 3.0 L in a reagent mode at a pulp temperature of 15 ± 1 °C, with a solid-to-liquid ratio of 1:6. During flotation enrichment, reagents that selectively interact with the minerals constituting shungite are used. In the work, we used kerosene TS-1—1134 g/t, and pine oil Flotol 5219—1260 g/t. The duration of the main and control flotation was 10 min. Additional stages of recleaning flotation were conducted in three phases to increase the carbon content in the concentrate. The enrichment scheme is shown in Figure 3.

### 2.3. Preparation of Rubber Compounds

The ingredients of the rubber compound were mixed in a closed laboratory Plasticorder internal mixer Lab. Station Plasti-Corder, W50 (Brabender, Duisburg, Germany) at a temperature of 60 °C and a rotor rotation speed of 60 rpm according to the standard formulation for nitrile butadiene rubber according to State Standard GOST 34754-2021* (* State Standard) (Table 1). The type and concentration of ingredients in the formulation are specified in the standard. The formulation chosen for the analysis was standard, not industrial, because industrial formulations usually contain a larger number of ingredients, which can make it difficult to assess the impact of the tested fillers on the properties of rubbers.

The ratios of the fillers are shown in Table 2.

### 2.4. Research Methods

#### 2.4.1. Determination of the Chemical Composition of Shungite Materials

The chemical composition of the shungite fillers was determined using an electron probe microanalyzer Superprobe-733 (JEOL, Tokyo, Japan) and an energy-dispersive spectrometer INCA Energy (Oxford Instruments, Oxfordshire, UK) at an accelerating voltage of 25 kV and a probe current of 25 nA.

#### 2.4.2. Determination of the Specific Surface Area of Fillers

The specific surface area of the fillers was determined by the method of thermodesorption of the gas adsorbate according to the BET theory using a “Sorbtemeter” (Katakon CJSC, Cheboksary, Russia) under the influence of liquid nitrogen on the test material.

#### 2.4.3. Determination of the Surface Morphology of Fillers

The surface morphology of ShO and ShC was studied with a scanning electron microscope Quanta 3D 200i Dual system (FEI, Hillsboro, OR, USA) at the National Nanotechnology Laboratory of Al-Farabi Kazakh National University.

#### 2.4.4. Determination of Mooney Viscosity

The Mooney viscosity of the rubber compounds was determined on a Mooney Viscozimeter-UGT7080S2 (Gotech, Taichung City, Taiwan) at a temperature of 100 °C (material pre-heating period 1 min, the rotor rotation duration 4 min).

#### 2.4.5. Study of the Cure Characteristics

The rheometric characteristics of the rubber compounds were determined using an MDR (Moving Die Rheometer) MD-3000A (Gotech, Taichung City, Taiwan) in accordance with State Standard GOST 12535-84 at a chamber temperature of 150 °C for 30 min.

#### 2.4.6. Vulcanization

The vulcanization process was carried out in a laboratory vulcanization press with induction heating of plates 100-400-2E (Krasin Plant CJSC, Kirov, Russia) at a temperature of 150 °C according to State Standard GOST 269-66.

#### 2.4.7. Determination of the Physical–Mechanical Properties of Vulcanizates

The physical–mechanical properties of the rubber samples were evaluated according to State Standard GOST 270-75 by tensile strength, elongation at break, and residual elongation, which were determined on the universal electromechanical testing machine TRM-P 50 C1 Tochline (NPO Tochpribor LLC, Rostov-on-Don, Russia). Shore A hardness of the samples was measured on a portable hardness tester TH-200 (Time Group, Beijing, China) according to State Standard GOST 263-75. Rebound resilience was determined on a Shoba-type rebound resilience tester UMR-1 (Polymermash Group LLC, St. Petersburg, Russia) according to State Standard GOST 27110-86.

The oil and petroleum resistance of the rubber samples was determined by the change in mass of samples immersed in standard petroleum liquid of SZhR-1 grade for 72 h at 23 °C according to the formula:

$$\alpha =\frac{m_{1}-m_{0}}{m_{0}}\times 100\%\;,$$

where, m_1_—mass of the swollen sample, m_0—_mass of the original sample.

#### 2.4.8. Determination of Crosslink Density

For the testing, six specimens from each rubber sample of different shapes (square (10 mm × 10 mm × 2 mm), triangle (10 mm × 10 mm × 10 mm × 2 mm), circle (diameter 10 mm, thickness 2 mm)) were prepared. The samples were weighed and placed in a glass vessel (separately for each vulcanizate) and immersed in tetrachloromethane (CCl₄). The experiments were conducted at room temperature for 7 days. After 7 days, the samples were removed, dried with filter paper, and weighed. The number-average molecular mass of the polymer chain segments between adjacent nodes of the vulcanizate network was determined using Equation (1) based on the Flory–Rehner theory [46].

(1) $$M_{c}=-\frac{\rho_{1}}{\rho_{2}}\cdot \frac{M_{2}\left(\varphi^{\frac{1}{3}}-\frac{2}{f\cdot \varphi_{1}}\right)}{\ln(1-\varphi )+\varphi +\chi \cdot \varphi^{2}}$$

where, *ρ*_1_, *ρ*_2_—density of nitrile butadiene rubber (0.96 g/cm^3^) and density of the solvent CCl_4_ (1.595 g/cm^3^); *χ*—polymer-solvent interaction parameter (0.478); *M*_2_—molecular weight of CCl_4_ (153.8 g/mol); *f*—functionality of network nodes (4); *φ*—volume fraction of elastomer in the swollen sample.

The experimentally determined value of the volume fraction of elastomer, denoted as φ, is calculated from the mass of the original (m_0_) and swollen (m_s_) elastomer samples using Equation (2).

(2) $$\varphi =\frac{1}{(1+(\frac{m_{s}}{m_{0}}-1)\cdot \frac{\rho_{1}}{\rho_{2}}}$$

## 3. Results and Discussion

For fillers used in the rubber industry, important parameters include chemical composition, specific surface area, size of primary aggregates, and structure, which together determine the intensity of interaction between the filler and rubber macromolecules. On the basis of the above-mentioned, in the first stage of the work, the differences in the surface morphology of the studied shungite fillers and their chemical compositions were determined.

**Figure 4:**
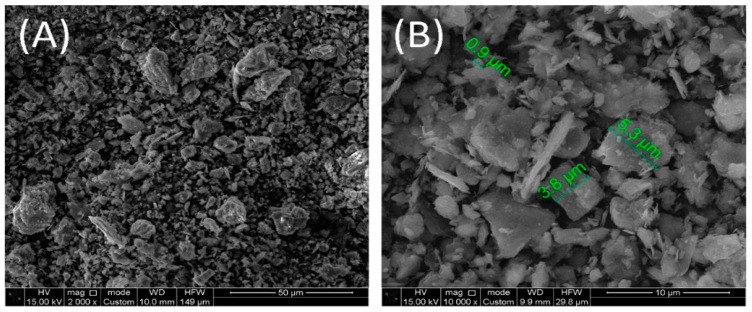
SEM images of ShO at different magnifications: (**A**) ×2000 and (**B**) ×10,000.

**Figure 5:**
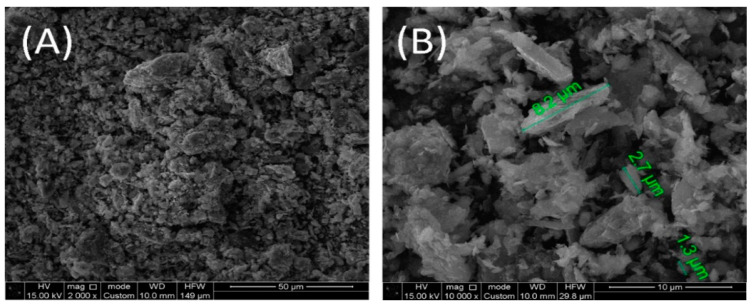
SEM images of ShC at different magnifications: (**A**) ×2000 and (**B**) ×10,000.

The surface morphology of ShO and ShC was studied by scanning electron microscopy (SEM) (Figure 4 and Figure 5). A detailed study of the images shows an insignificant difference between the sizes of crystalline particles of the shungite fillers, which mainly have oblong shapes. This may influence the flow properties of the compound and the degree of interaction between the fillers and the rubber macromolecules.

The most important factor determining the reinforcing effect of fillers is their dispersity, characterized by particle size or specific surface area. Fillers with particle sizes ranging from 5 to 1000 nm can be used to reinforce rubber, and fillers with particle sizes from 10 to 50 nm have the greatest reinforcing properties. This is confirmed by the results of the work [47,48].

The results of the X-ray spectral analysis of shungite fillers indicated (Table 3) that the main components of ShO and ShC are carbon, silicon dioxide, and aluminum oxide. During the flotation process for obtaining ShC, the carbon content increased more than 3.5 times (from 11 wt% to 39 wt%), while the proportion of silicon dioxide decreased more than 3 times (from 49.44 wt% to 13.64 wt%). The content of aluminum oxide practically did not change (21.13 wt% and 20.26 wt%, respectively). The proportion of iron oxide increased by 30% (from 6.75 wt% to 9.37 wt%) during the flotation of ShO. The amounts of K, Ca, Ti, and Mn oxides changed less than 1%.

The analysis of literature data on the influence of the chemical composition of mineral fillers on the physical–mechanical properties of rubbers suggests the following. Increasing the carbon content in ShC (39 wt%) enhances the mechanical properties of rubbers, such as compressive strength and elasticity. These parameters are of particular importance for rubbers that need to retain properties over a wide range of operating temperatures. The high content of SiO_2_ in ShO (49.44 wt%) contributes to increased strength and wear resistance, which is important for rubber applications under friction and stress [49]. The reduction of SiO_2_ in ShC (13.64 wt%) may limit some of the positive effects. Aluminum oxide (Al_2_O_3_) increases the heat resistance and tensile strength of rubbers [50,51]. The relatively stable content of Al_2_O_3_ in ShO and ShC (21.13 wt% and 20.26 wt%, respectively) allows us to expect good strength characteristics of rubbers during thermal aging. The oxides of several metals (Na_2_O, CaO, MgO, K_2_O) may act as vulcanization activators [52]. Each of these factors may ultimately affect the technological and operational properties of the resulting rubber products.

During the enrichment of ShO, an increase in the specific surface area of the mineral material was observed. The specific surface area of ShO and ShC was 9 m^2^/g and 17 m^2^/g, respectively (Table 3). The specific surface area of shungite affects the vulcanization process in the following way: the developed surface of shungite leads to increased sorption of the rubber compound’s ingredients, including the vulcanizing group. This prevents the formation of a sufficiently dense and strong vulcanization network and, consequently, reduces the strength characteristics of vulcanizates [49].

**Figure 6:**
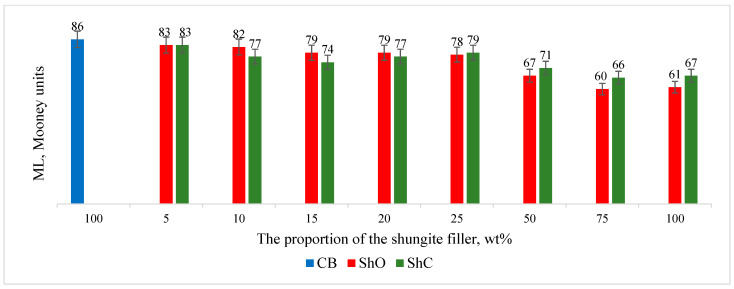
The influence of the proportion of the shungite filler (wt%) on Mooney viscosity of the rubber compounds.

Viscosity is considered an important characteristic affecting the uniformity of ingredient distribution in a rubber compound. It was found that at partial or complete replacement of CB with ShO and ShC in the rubber compound, there is a decrease in Mooney viscosity ML (1 + 4) 100 °C in comparison with the standard sample filled only with CB (Figure 6).

When replacing less than 50 wt% of CB with the shungite fillers, no significant difference in viscosity between ShO and ShC was observed. However, when replacing more than 50 wt% of CB with the shungite fillers, the viscosity of the samples decreases to 61–70 Mooney units (Figure 6). At the same time, the viscosity of the rubber compound containing ShC is higher by 4–6 Mooney units. That is, the use of ShO and ShC leads to an increase in the plasticity of the rubber compounds, which in turn facilitates processing and allows for a reduction in the share of plasticizer. This is probably due to the presence of some fullerenes in the composition of the shungite fillers, which act as a kind of “lubricant” [53].

The analysis of the curing characteristics of the rubber compounds showed that regardless of the differences in the chemical composition of the shungite fillers, the introduction of both ShO and ShC leads to an increase in the scorch (*t_s_*) and cure (*t*_90_) times (Table 4).

Apparently, the decrease in the vulcanization rate is due to the adsorption of the accelerator, activators, and other components of the vulcanizing group on the surface of the shungite fillers. This leads to the need to adjust the formulations to increase the content of the vulcanizing group.

**Scheme 1:**
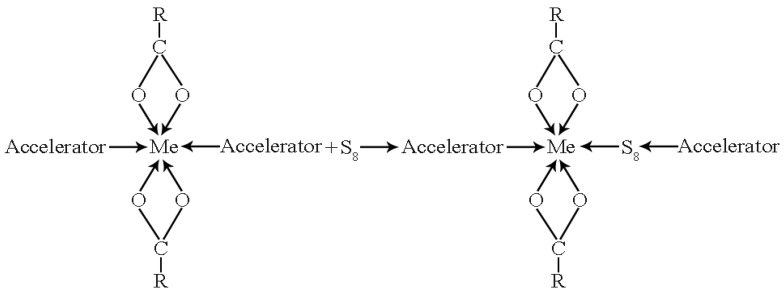
Coordination complexes of the vulcanizing group (sulfur, accelerator, activator).

In addition, the slowing of the vulcanization process may be due to the influence of metal ions contained in the shungite filler (Na^+^, Mg^+2^, Ca^+2^, K^+^). It is known that in a complex system consisting of rubber, sulfur, accelerator, and activator, coordination interactions occur, leading to the formation of intermediate components with various types of bonds. Coordination complexes are better dissolved in rubber than all initial components, which provides better distribution in the rubber matrix and more uniform vulcanization. This manifests the activation of the process. The structure of the complexes depends on the type of accelerator, but in general, it can be represented by the following Scheme 1 [54].

Metal ions present in the shungite filler can form less active complexes with the vulcanization accelerator that compete with zinc oxide.

The values of the maximum torque (T_max_) of the samples containing ShC and ShO decrease with the increase in their proportion in the filler composition, which correlates with the obtained values of Mooney viscosity of the studied samples (Figure 6, Table 4). This result once again highlights the influence of the metal oxides present in the composition of the shungite fillers on the vulcanization process, as noted in several studies [55,56,57,58].

**Figure 7:**
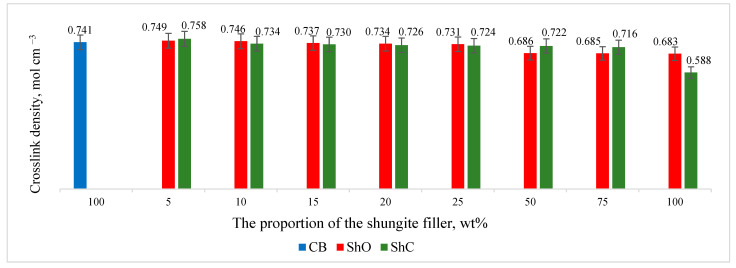
Influence of the proportion of the shungite filler on crosslink density of the vulcanizates.

The results of determining the density of the network chains of the samples (Figure 7) containing the shungite fillers indicated, as expected from the results of rheometric tests, the formation of a more sparse three-dimensional network when replacing more than 5–10 wt% of CB with the shungite fillers. These data are consistent with the physical–mechanical properties of the rubbers presented in Table 5.

**Figure 8:**
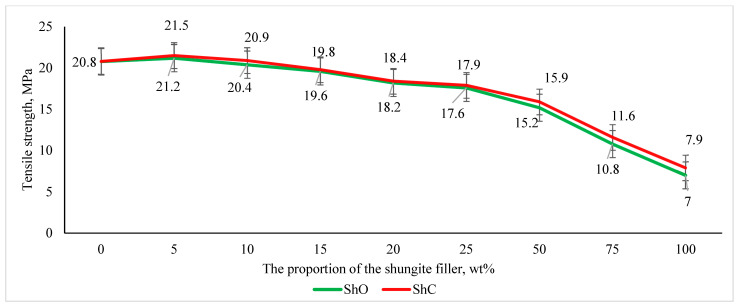
Influence of the proportion of the shungite filler on tensile strength.

The tensile strength of the samples in which 5–10 wt% of CB was replaced by ShC and ShO (samples 2–3, 10–11) remains at the level of the control sample (Figure 8). Further increase in the amount of the shungite fillers leads to a decrease in this parameter. The decrease in strength characteristics may be due to the fact that the shungite fillers, having their unique texture and structure, may not provide the same degree of compaction in the rubber composite as the traditional filler CB, which leads to a lower tensile strength of the rubber. Replacing 75 wt% of CB with ShO and ShC and completely replacing CB with ShO and ShC resulted in a dramatic decrease in tensile strength of the rubbers to 10.8 and 7.0 MPa for ShO, and to 11.6 and 7.9 MPa for ShC. The differences in tensile strength between the rubbers containing ShO and ShC indicate that the concentration of shungite ore to increase the carbon content positively affects the properties of the rubber. In general, the decrease in tensile strength of the samples with an increase in the proportion of the shungite filler by more than 10 wt% in the formulation is probably due to a decrease in the density of the vulcanization network chains of the samples, as well as a decrease in the total amount of the filler—carbon black.

**Figure 9:**
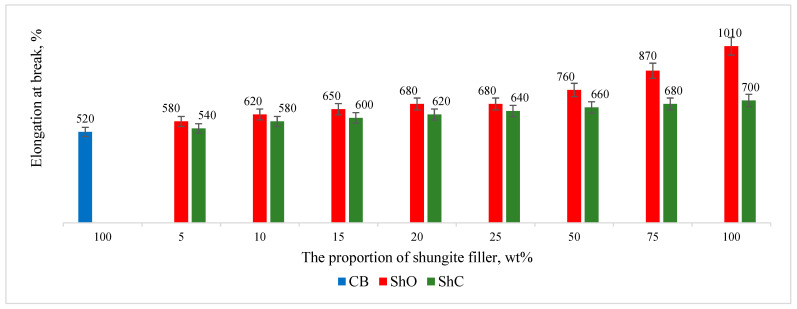
Influence of the proportion of the shungite filler on elongation at break.

It is logical that the elongation at break of the samples increases with increasing the content of the shungite fillers. Complete replacement of CB with ShO (sample 9, Table 5, Figure 9) leads to an increase in elongation at break by 94%, whereas complete replacement of CB with ShC (sample 17, Table 5, Figure 9) leads to an increase of 30% in comparison with the control sample. This correlates with the data on tensile strength and is associated with the change in the degree of crosslinking of the samples. Thus, the concentration of ShO leads to a lower dependence of elongation at break on the proportion of the shungite filler. The obtained data do not contradict the results of studies published by the authors [25,26].

The results of the determination of the residual elongation of the rubber samples are of particular interest. They show that with increasing the amount of the shungite fillers, the residual elongation of the samples also increases (Table 5). This is likely related to the influence of the chemical composition of ShO and ShC on the vulcanization network of the rubbers. It is important to note that the samples filled with ShO exhibit higher residual elongation compared to those filled with ShC (samples 2–9, Table 5). Consequently, rubbers filled with ShC possess better properties.

**Figure 10:**
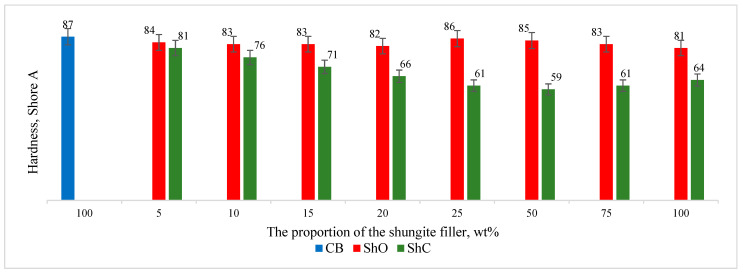
Influence of the proportion of the shungite filler on Shore A hardness.

Upon partial or complete replacement of CB with ShO, a slight decrease in Shore A hardness (samples 2–9, Figure 10) is observed compared to the control sample filled with CB (sample 1, Table 5, Figure 10). As evident from the presented data, as the proportion of ShC in the filler increases from 5 wt% to 25 wt% (samples 5–14, Table 5, Figure 10), the Shore A hardness decreases linearly from 81 to 61 units. When the proportion of ShC is increased to 50 wt% or more, as well as in the case of complete replacement of CB with ShC (samples 16–17, Table 5, Figure 10), the hardness increases slightly. This can be explained by the fact that with an increase in the ShC content, the amount of silicon dioxide in the rubber matrix increases, which correlates with the data on the influence of SiO_2_ on the hardness of rubbers [59].

**Figure 11:**
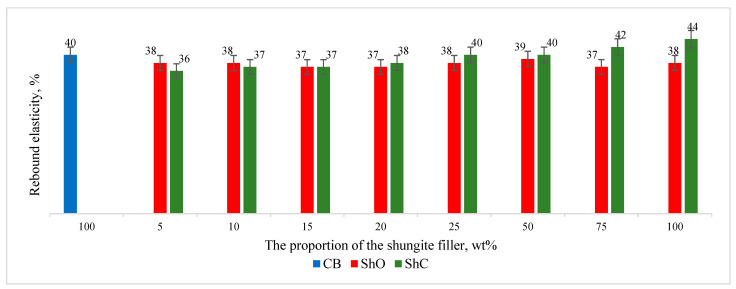
Influence of the proportion of the shungite filler on rebound elasticity.

According to the results of the study, the shungite fillers do not have a significant effect on rebound elasticity compared to the control sample filled with CB (Figure 11).

When determining the resistance of rubbers to aggressive media, it was found that after exposure of the samples with different contents of ShO and ShC in the petroleum liquid of SZhR-1 grade for 72 h at 23 ± 1 °C, the change in mass does not exceed +0.5%. This allows us to assert that ShO and ShC do not impair the resistance of rubbers to SZhR-1. The results obtained from the testing of samples in which up to 10 wt% of CB was replaced with ShO and ShC correlate with the data from the study by Ushmarin et al. [35], in which Kazakhstan’s shungite of the brand “Taurite TS-D” was used.

## 4. Conclusions

The influence of natural carbon-containing fillers—shungite ore (ShO) (the Bakyrchik deposit, Kazakhstan) and shungite concentrate (ShC), obtained through a five-stage flotation enrichment process—on the properties of elastomer composites based on nitrile butadiene rubber of BNKS-28 AMN grade has been investigated. It was found that replacing carbon black (CB) with ShO and ShC reduces Mooney viscosity ML (1 + 4) 100 °C by up to 29% compared to the standard sample filled only with CB. Additionally, the viscosity of the rubber compound containing ShC was 4–6 Mooney units higher. Rheometric analysis of the rubber compounds revealed that the use of the shungite fillers increased scorch time (t_s_) by up to 36% and cure time (t_90_) by up to 35%.

The main difference in the shungite fillers is that in the chemical composition of ShC, the carbon content is 3.5 times higher than in ShO (39 wt% and 11 wt%, respectively), but silicon oxide is 3 times lower (49.4 wt% and 13.6 wt%, respectively).

Complete replacement of CB with the shungite fillers reduces tensile strength of the rubbers by up to 66%. In the case of ShO, elongation at break increases by 94%, and in the case of ShC—by 30% compared to the control sample filled with CB. As the proportion of the shungite fillers increases, residual elongation of the samples rises, Shore A hardness decreases by up to 26%, and rebound elasticity remains at the control level. Complete replacement of CB with the shungite fillers results in a mass change of up to +0.5% after 72 h of exposure in the petroleum liquid of SZhR-1 grade at room temperature. The best results were obtained for the samples where 5–10 wt% of CB was replaced with the shungite fillers. At this ratio, it was possible to maintain tensile strength at the level of the control sample, and the change in mass of the samples after 72 h of exposure in SZhR-1 at 23 ± 2 °C was no more than +0.1%.

## Figures and Tables

**Table 1:** Formulation of the rubber compound.

No	Ingredients	Content, phr **
1	BNKS-28 AMN	100.0
2	Sulfur	1.5
3	ZnO	5.0
4	Stearic acid	1.5
5	MBTS	1.5
6	Filler	45.0

** Parts per hundred of rubber.

**Table 2:** The ratio of the fillers in the formulation of the rubber compound.

No	Quantity, wt%		
	CB P 324	ShO	ShC
1 (control)	100	0	0
2	95	5	0
3	90	10	0
4	85	15	0
5	80	20	0
6	75	25	0
7	50	50	0
8	25	75	0
9	0	100	0
10	95	0	5
11	90	0	10
12	85	0	15
13	80	0	20
14	75	0	25
15	50	0	50
16	25	0	75
17	0	0	100

**Table 3:** Results of the X-ray spectral analysis of the shungite fillers.

Chemical Element, Component	Content, wt%	
	Shungite Ore	Shungite Concentrate
C	11.0 ± 0.5	39.0 ± 0.5
Na_2_O	1.77 ± 0.1	1.39 ± 0.1
MgO	1.31 ± 0.1	1.57 ± 0.1
Al_2_O_3_	21.13 ± 0.1	20.26 ± 0.1
SiO_2_	49.44 ± 0.1	13.64 ± 0.1
P_2_O_5_	0.22 ± 0.01	<0.10 ± 0.01
K_2_O	3.42 ± 0.1	3.50 ± 0.1
CaO	2.47 ± 0.1	1.16 ± 0.1
TiO_2_	3.06 ± 0.1	2.17 ± 0.1
MnO	0.20 ± 0.01	<0.10 ± 0.01
Fe_2_O_3_	6.75 ± 0.1	9.37
Specific surface area, m^2^/g	9	17

**Table 4:** Parameters of the rheometric curves of vulcanization.

Composition, No.	t_s_, min	T_min_, dN·m	T_max_, dN·m	t_(90)_, min
1	1.8	8	41	13.8
2	2.4	9	45	15.5
3	2.5	10	44	15.7
4	2.5	10	43	15.7
5	2.4	9	42	15.6
6	2.5	9	40	15.6
7	2.5	8	36	16.0
8	2.5	8	33	16.8
9	3.8	9	31	18.7
10	2.0	12	50	15.0
11	2.1	11	46	16.0
12	2.0	11	45	15.8
13	2.1	12	46	16.1
14	2.2	12	44	16.2
15	2.4	8	29	16.6
16	2.5	8	29	16.8
17	3.2	9	33	18.2

**Table 5:** Results of the testing of vulcanizates.

No.	Composition Ratios (CB:ShO:ShC)	*ε*, %	*ε’*, %	∆m, %:
1	100:0:0	520	18	+0.1
2	95:5:0	580	18	+0.1
3	90:10:0	620	20	+0.1
4	85:15:0	650	20	+0.1
5	80:20:0	680	22	+0.1
6	75:25:0	680	23	+0.1
7	50:50:0	760	25	+0.3
8	25:75:0	870	37	+0.1
9	0:100:0	1010	49	+0.4
10	95:0:5	540	16	+0.1
11	90:0:10	580	19	+0.1
12	85:0:15	600	20	+0.1
13	80:0:20	620	22	+0.2
14	75:0:25	640	23	+0.2
15	50:0:50	660	27	+0.3
16	25:0:75	680	30	+0.4
17	0:0:100	700	32	+0.5

Note: *ε*, %—elongation at break; *ε’*, %—residual elongation; ∆m, %—change in mass in the petroleum liquid of SZhR-1 grade.

## Data Availability

Data are contained within the article.
